# Synergistic Antinociceptive Effect of β-Caryophyllene Oxide in Combination with Paracetamol, and the Corresponding Gastroprotective Activity

**DOI:** 10.3390/biomedicines12051037

**Published:** 2024-05-08

**Authors:** Josué Vidal Espinosa-Juárez, Jesús Arrieta, Alfredo Briones-Aranda, Leticia Cruz-Antonio, Yaraset López-Lorenzo, María Elena Sánchez-Mendoza

**Affiliations:** 1 Escuela de Ciencias Químicas, Universidad Autónoma de Chiapas, Ocozocoautla de Espinosa 29140, Chiapas, Mexico; josue.espinosa@unach.mx; 2 Laboratorio de Farmacología de Plantas Medicinales Mexicanas, Escuela Superior de Medicina, Instituto Politécnico Nacional, Plan de San Luis y Díaz Mirón, Colonia Casco de Santo Tomás, Miguel Hidalgo, Mexico City 11340, Mexico; jarrietav@ipn.mx (J.A.); yarlop_2310@outlook.com (Y.L.-L.); 3 Laboratorio de Farmacología, Facultad de Medicina Humana, Universidad Autónoma de Chiapas, Tuxtla Gutiérrez 29050, Chiapas, Mexico; alfred725@hotmail.com; 4 Facultad de Estudios Superiores Zaragoza, Universidad Nacional Autónoma de México, Av. Guelatao No. 66, Colonia Ejército de Oriente, Iztapalapa, Mexico City 09230, Mexico; letycruza@yahoo.com.mx

**Keywords:** β-caryophyllene oxide, paracetamol, drug interaction, anti-nociception, gastroprotection

## Abstract

Pain is the most frequent symptom of disease. In treating pain, a lower incidence of adverse effects is found for paracetamol versus other non-steroidal anti-inflammatory drugs. Nevertheless, paracetamol can trigger side effects when taken regularly. Combined therapy is a common way of lowering the dose of a drug and thus of reducing adverse reactions. Since β-caryophyllene oxide (a natural bicyclic sesquiterpene) is known to produce an analgesic effect, this study aimed to determine the anti-nociceptive and gastroprotective activity of administering the combination of paracetamol plus β-caryophyllene oxide to CD1 mice. Anti-nociception was evaluated with the formalin model and gastroprotection with the model of ethanol-induced gastric lesions. According to the isobolographic analysis, the anti-nociceptive interaction of paracetamol and β-caryophyllene oxide was synergistic. Various pain-related pathways were explored for their possible participation in the mechanism of action of the anti-nociceptive effect of β-caryophyllene oxide, finding that NO, opioid receptors, serotonin receptors, and K^+^_ATP_ channels are not involved. The combined treatment showed gastroprotective activity against ethanol-induced gastric damage. Hence, the synergistic anti-nociceptive effect of combining paracetamol with β-caryophyllene oxide could be advantageous for the management of inflammatory pain, and the gastroprotective activity should help to protect against the adverse effects of chronic use.

## 1. Introduction

Pain has been defined by the International Association for the Study of Pain (IASP) [1] in an interesting and all-encompassing manner. The IASP refers to two dimensions of pain, the physical sensation of intensity and the emotional experience of unpleasantness. Moreover, their definition of pain includes its relation to real or potential tissue damage. The two types of pain identified, acute and chronic, are distinguished by their temporal relationship to an injury or other tissue damage [2]. Acute pain is expected to be of limited duration, while chronic pain persists at least 3 months [3]. Chronic pain is classified as nociceptive, neuropathic, or nociplastic [3,4]. 

Acute pain is managed pharmacologically by means of nonselective non-steroidal anti-inflammatory drugs (NSAIDs), selective cyclooxygenase-2 inhibitors (COX-2), opioids, or a combination of drugs (e.g., an NSAID plus an opioid) [5]. Similar treatment options are employed for patients with chronic nociceptive pain. Chronic neuropathic pain is handled with opioids plus paracetamol, followed by drugs that act on the central nervous system (e.g., antidepressants and antiepileptic drugs). Drugs commonly used for the treatment of nociplastic pain are pregabalin and duloxetine [3]. 

Both acute and chronic pain can be treated by multimodal analgesia, which is a pharmacological strategy aimed at providing pain relief [6,7] by combining two or more medications from distinct pharmacological groups [7]. Such a combination may lead to greater effectiveness, lower doses, and/or reduced side effects, which are all key components of the pharmacological treatment of pain [6]. 

Paracetamol, also called acetaminophen, is an analgesic and antipyretic drug that shows efficacy for mild-to-moderate pain [8]. It is considered the most commonly used over-the-counter drug in the world [9], being prescribed to children, adults, and elderly people. Paracetamol is particularly advantageous for patients when NSAIDs are contraindicated, for those with gastric ulcers or bronchial asthma, and for pregnant women and lactating mothers [10]. It has recently been administered for the treatment of severe pain [8] and is included in the World Health Organization list of essential medicines for the management of the pain and palliative care of cancer patients [11]. 

Paracetamol is combined with other drugs to treat both acute and chronic pain [6,7]. For example, its combination with ibuprofen produces enhanced analgesia compared to its administration alone [7]. Among the multimodal applications of paracetamol for chronic pain is its combination with tramadol (an opioid) to treat arthritis and fibromyalgia [6]. This combination may, in some cases, lead to adverse effects such as hepatotoxicity [8]. 

To reduce the exposure of patients to synthetic drugs, there is an ongoing search for natural products with analgesic activity and limited side effects [12]. The combination of paracetamol with the ethanol extract of *Bidens odorata* [13] or with pharmaceutical formulations (tincture and syrup) containing *Thymus vulgaris* L. [14] produces a synergistic antinociceptive effect and potentiation interaction, respectively.

β-caryophyllene oxide, the oxidation derivative of caryophyllene, is a natural bicyclic sesquiterpene found in the essential oils of several plants, including *Ocimum* spp., *Salvia glutinosa*, *Syzygium cordatum* [12], *Rosmarinus officinalis*, *Piper nigrum*, and *Cannabis sativa* [15]. As a result of its approval as a flavoring by the Food and Drug Administration (FDA) and the European Food Safety Authority (EFSA), it is used as an additive in food and cosmetics [12]. It is reported to have antioxidant, antiviral, anticancer, anti-inflammatory, and analgesic properties [12,16]. 

A potential synergistic analgesic effect of paracetamol and β-caryophyllene oxide is worth analyzing, considering the analgesic activity of both compounds and the successful synergistic combination of paracetamol with some other compounds to provide analgesic effects [6,7]. Hence, the aim of the current contribution was to evaluate the anti-nociceptive effect of the treatment of mice (in the formalin model) with a combination of paracetamol and β-caryophyllene oxide. The formalin model was also utilized to explore the possible involvement of various pain-related pathways in the mechanism of action of β-caryophyllene oxide. Additionally, the gastroprotective activity of the combination was determined in a model of ethanol-induced gastric lesions in mice. 

## 2. Materials and Methods

### 2.1. Animals

Male CD1 mice (weighing 20–30 g) were maintained on a 12 h light/dark cycle, with food and water provided ad libitum. While water was available throughout the experiment, food was removed twelve hours prior to the evaluations, whether for nociception or gastroprotection. The animals were obtained from the Facultad de Medicina Humana of the Universidad Autónoma de Chiapas. Mice destined for the assessment of gastroprotection were placed in individual cages with a mesh floor twelve hours before the test. All experimental protocols followed the ethical guidelines for pain experimentation on conscious animals [17] and the regulations established in the Mexican Official Norm (The Technical Specifications for the Production, Care, and Use of Laboratory Animals, NOM-062-ZOO-1999) [18]. The experimental procedure was approved by the Institutional Committee of the Universidad Autónoma de Chiapas (approval number 03/ECQ/RPR/137/23). There were six animals per group and each animal was used only once. At the end of the experiment, the mice were euthanized.

### 2.2. Drugs

Paracetamol, β-caryophyllene oxide, methiothepin, glibenclamide, [1,2,4] oxadiazolo [4,3-a]quinoxalin-1-one (ODQ), N(G)-nitro-l-arginine methyl ester hydrochloride (l-NAME), and l-arginine were obtained from Sigma-Aldrich (St. Louis, MO, USA), and naloxone was acquired from Pisa Laboratories (Mexico City, Mexico). All drugs were suspended in carboxymethylcellulose (0.1%) and prepared minutes before being given to the animals. β-caryophyllene oxide and paracetamol were administered orally (0.1 mL/10 g of body weight). Naloxone, methiothepin, l-NAME, ODQ, l-arginine, and glibenclamide were injected intraperitoneally (0.1 mL/10 g of body weight). Formalin (2%) was prepared by diluting aqueous formaldehyde to 37% from J.T. Baker (Matsonford Rd. Radnor Township, PA, USA).

### 2.3. Anti-Nociceptive Evaluation

The anti-nociceptive effect of the treatments was assessed with the formalin test, as previously described [19]. Briefly, after applying 20 µL of formalin (2%) to the dorsal surface of the right hind paw of the rodent, the number of flinches was counted for 1 min. This one-minute determination was performed every 5 min over a 45 min period. The results of the observations were divided into two phases, the first constituted by minutes 0–10 and the second by minutes 15–45. The three treatments, administered orally 30 min prior to the application of formalin, consisted of paracetamol, β-caryophyllene oxide, and the combination of the two compounds. The drugs were administered by one researcher, and then another did the pharmacological evaluations in a blinded manner. Paracetamol was tested with doses of 10, 30, 100, and 300 mg/kg, and β-caryophyllene oxide with 3, 10, 30, and 100 mg/kg. The doses for the combination of paracetamol and β-caryophyllene oxide (1:1) are listed in Table 1. The effects of the doses of each treatment were examined by plotting the number of formalin-induced flinches as a function of time. The anti-nociceptive effect was depicted as the area under the curve (AUC), calculated by the trapezoidal method [20]. The percentage of anti-nociception was found by means of the following formula:

$$\%Antinociception=\frac{AUC\left(control\;group\right)-AUC(test\;drug)}{AUC(control\;group)}\times 100$$

The mean effective dose (ED_50_) was calculated with a four-parameter Hill equation on GraphPad Prism software (version 8, SPSS Inc., Chicago, IL, USA).

### 2.4. Evaluation of the Type of Interaction of the Compounds in the Combination Treatment

An isobolographic analysis was constructed by using the mean effective dose (ED_50_) of the combination and individual treatments, utilizing the methodology described by Tallarida (2000) [21], with the ED_50_ of paracetamol placed on the *y*-axis and the ED_50_ of β-caryophyllene oxide on the *x*-axis. Subsequently, the additivity line was drawn, the midpoint of which served to identify the theoretical ED_50_. Based on the individual ED_50_ of each drug, four doses were determined to test the combination treatment with the formalin model. The dose–response curve was constructed, and the experimental ED_50_ was calculated and compared to the theoretical ED_50_. Furthermore, the interaction index was found using the methodology of Tallarida (2002) [22]. The interaction is considered to be antagonistic with a value greater than 1, and synergistic with a value less than 1.

### 2.5. Evaluation of the Mechanism of Action of β-Caryophyllene Oxide 

Various pain-related pathways were investigated to explore the possible mechanism of action of the anti-nociceptive effect of β-caryophyllene oxide. The vehicle and all compounds related to the study of pain-related pathways were administered to the mice 15 min [19] before giving them β-caryophyllene oxide (30 mg/kg, p.o.). The compounds for the pretreatments were the following: naloxone (5 mg/kg, i.p.), a non–selective opioid receptor antagonist; methiothepin (1.0 mg/kg, i.p.), a non-selective serotonin receptor antagonist; l-arginine (100 mg/kg, i.p.), a nitric oxide (NO) precursor; l-NAME (10 mg/kg, i.p.), a non-selective nitric oxide synthase (NOS) enzyme inhibitor; ODQ (0.1 mg/kg, i.p.), a soluble guanylate cyclase inhibitor used to examine the involvement of NO; and glibenclamide (10 mg/kg, i.p.), a blocker of K^+^_ATP_ channels. The doses and treatment schedules utilized to explore the mechanisms of action were taken from previous reports [23,24,25,26]. After applying the pretreatments and β-caryophyllene oxide, the anti-nociceptive activity was evaluated and compared to that found with β-caryophyllene oxide alone.

### 2.6. Determination of the Protective Effect of the Combination Treatment on the Gastric Mucosa

Mice were divided into 6 groups (*n* = 6), and all the treatments were given orally at 0.1 mL/10 g of body weight. β-caryophyllene oxide was administered at 10.4 and 100 mg/kg to groups 1 and 2, respectively. Paracetamol was applied at 34.9 and 300 mg/kg to groups 3 and 4, respectively. Group 5 received the experimental ED_50_ of the combination treatment: β-caryophyllene oxide at 10.4 mg/kg plus paracetamol at 34.9 mg/kg. The vehicle was used for group 6.

Thirty minutes after giving the treatments to the animals, 0.2 mL of ethanol was administered orally, and 2 h later, the animals were sacrificed in a CO_2_ chamber. Their stomachs were dissected, filled with 2% formalin, and then submerged in formalin for 5 min. Subsequently, they were opened along the greater curvature to measure the gastric lesions under a stereoscopic microscope equipped with an ocular micrometer. The ulcer index was calculated as the sum of all lesions (area in mm^2^) in the stomach of each animal [27]. The percentage of gastroprotection was then determined with the following formula:

$$\%Gastroprotection=\frac{UIC-UIT}{UIC}\times 100$$

where UIC is the average ulcer index of the control group, and UIT is the average ulcer index of the experimental group for a given treatment [27].

### 2.7. Statistical Analysis

Data are expressed as the mean ± standard error of 6 animals per group. Firstly, the Shapiro–Wilk test was performed to ascertain whether the distribution of the data was normal or non-normal. In the former case, a one-way analysis of variance (ANOVA) was carried out for comparisons between multiple groups, followed by Dunnett’s post hoc test. For data with non-normal distribution, the Kruskal–Wallis test was employed, followed by Dunn’s multiple comparison. The theoretical ED_50_ of the isobologram was compared to the experimental ED_50_ with Student’s *t*-test. Statistical significance was considered at *p* < 0.05.

## 3. Results

### 3.1. Anti-Nociceptive Effect of Paracetamol and β-Caryophyllene Oxide

**Figure 1:**
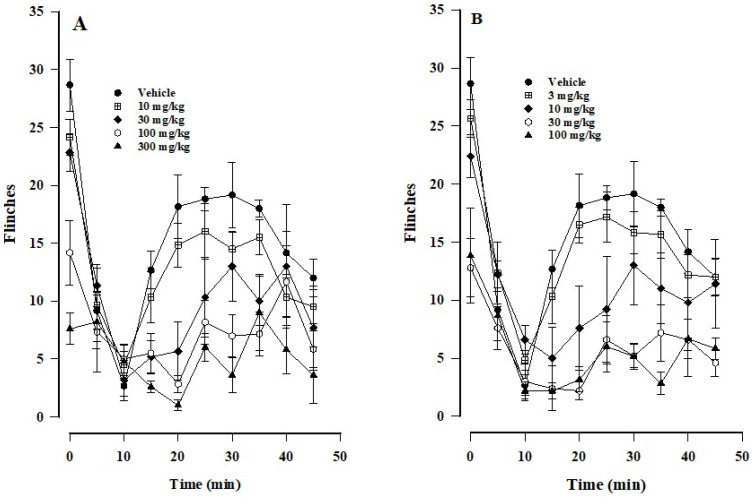
The time course of the anti-nociceptive effect of paracetamol (**A**) and β-caryophyllene oxide (**B**), manifested as a lower number of flinches (versus the control) in the model of formalin-induced pain. Data are expressed as the mean ± SEM of six assays.

The anti-nociceptive effect of the treatments was manifested as a lower number of flinches of the animals over time (in response to the application of 2% formalin) compared to the control group (Figure 1A,B). The formalin test produces a biphasic response, which is characteristic of inflammatory pain. Phase I represents a response to the initial stimulus and the resulting neurogenic pain, while phase II is characterized by inflammation and the pain stemming from tissue damage [28]. 

**Figure 2:**
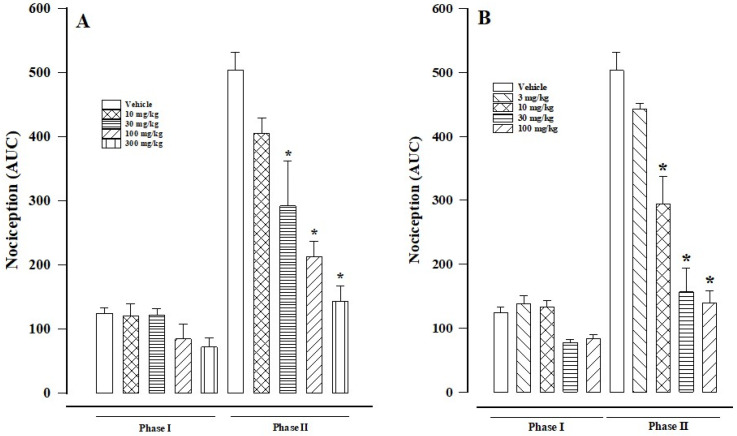
The AUC values are shown for phase I and phase II of the formalin test in relation to the different doses of paracetamol (**A**) and β-caryophyllene oxide (**B**). Data are expressed as the mean ± SEM of six assays. Statistical significance was assessed with the Kruskal–Wallis test, followed by Dunn’s multiple comparison (* *p* < 0.05 versus the control).

There was a greater response to pain in the vehicle control group than the groups treated either with paracetamol (Figure 1A) or β-caryophyllene oxide (Figure 1B). Nociception was expressed as the AUC of a treatment (Figure 2A,B), calculated for each phase. As can be appreciated, the drugs both exerted a significant anti-nociceptive effect in phase II but not in phase I.

Paracetamol produced a dose-dependent reduction in nociceptive behavior in phase II. The percentages of anti-nociception at the doses of 10, 30, 100, and 300 mg/kg were 19.45, 41.97, 57.78, and 71.68%, respectively. There was no significant difference (versus the control) at the lowest dose (10 mg/kg), but a significant difference did indeed exist at the higher doses (30, 100, and 300 mg/kg; Figure 1A).

Although the level of anti-nociception was not significant for β-caryophyllene oxide (versus the control) at 3 mg/kg (13.77% anti-nociception), it was indeed significant at the doses of 10, 30, and 100 mg/kg (41.58, 68.90, and 72.58% anti-nociception, respectively). Interestingly, the maximum response was practically the same at the 30 and 100 mg/kg dose (except at 35 min; Figure 1B and Figure 2B).

**Figure 3:**
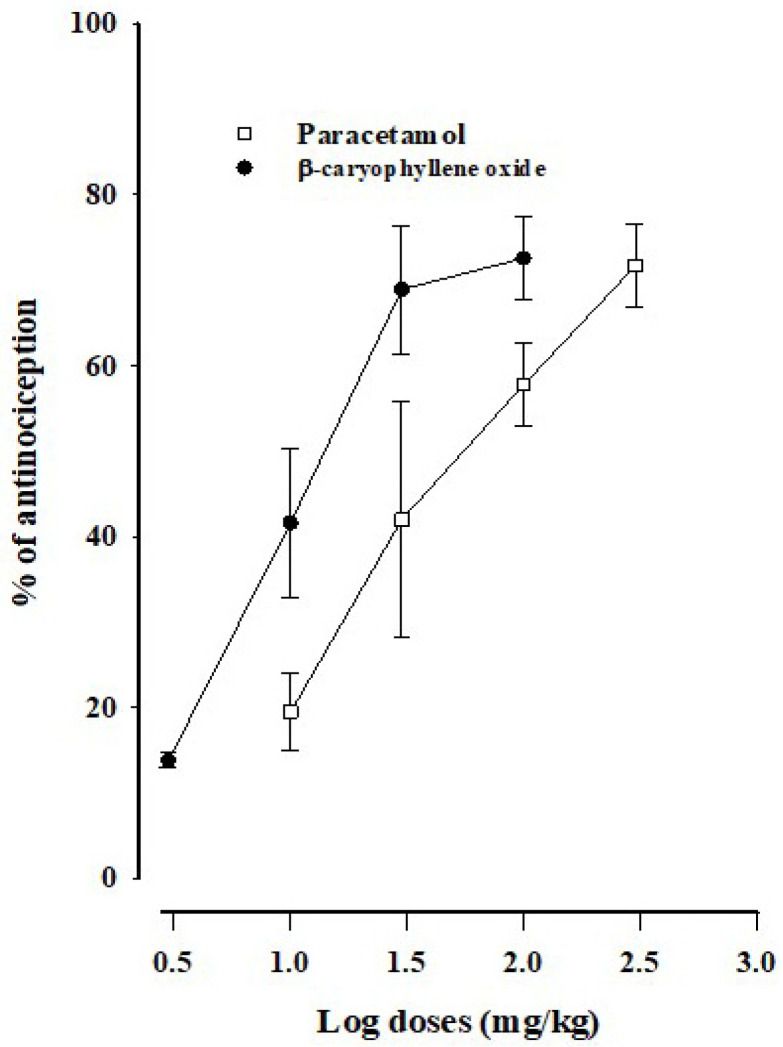
Log dose–response curves (DRCs) for the anti-nociception produced by paracetamol and β-caryophyllene oxide in the formalin test. Data are expressed as the mean ± SEM of six assays.

According to the dose–response curves of paracetamol and β-caryophyllene oxide (Figure 3), both drugs have a very similar efficacy at the highest doses, being 71.68 ± 4.85% for paracetamol and 72.58 ± 4.78% for β-caryophyllene oxide. However, β-caryophyllene oxide is more potent than paracetamol, as evidenced by the shift to the right of the paracetamol dose–response curve with respect to the β-caryophyllene oxide curve. The ED_50_ values are 29.67 ± 1.17 mg/kg for β-caryophyllene oxide and 98.17 ± 4.73 mg/kg for paracetamol.

### 3.2. Anti-Nociceptive Effect of the Combination Treatment

**Figure 4:**
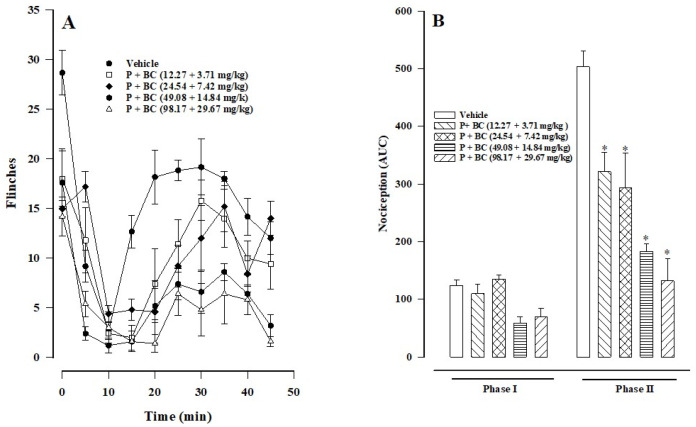
(**A**) The time course of the anti-nociceptive effect of the combination treatment (paracetamol (P) plus β-caryophyllene oxide (BC)) in phases I and II of the formalin test. (**B**) The AUC of various doses of the combination treatment in phases I and II. Data are expressed as the mean ± SEM of six assays. Statistical significance of the treatment versus control group was evaluated with one-way ANOVA, followed by Dunnett’s post hoc test (* *p* < 0.05).

As the dose of the combination of paracetamol and β-caryophyllene oxide (in a 1:1 proportion) increased (Figure 4A), nociception decreased in phase II, observing a dose-dependent effect. The vehicle control group showed the greatest nociceptive effect. Only during phase II was there a significant difference between the AUC values of the control group and the combination treatment (at distinct doses; Figure 4B). The anti-nociception generated by the highest to the lowest dose of the combination treatment was 73.77, 63.64, 41.58, and 36.12% (Table 1, 1–4).

**Figure 5:**
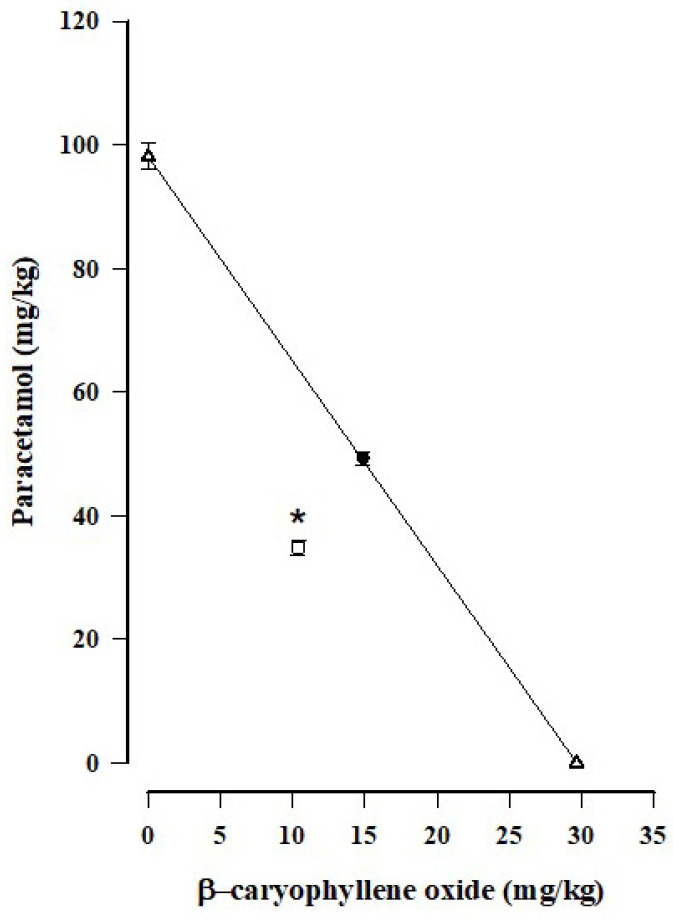
Isobologram of the interaction between paracetamol and β-caryophyllene oxide in the combination treatment, tested on mice by using the formalin model. The ED_50_ values of the individual treatments with β-caryophyllene oxide and paracetamol were plotted. The line connecting these two ED_50_ values represents the theoretical additive effect, and the point in the middle of this line is the theoretical additive ED_50_ (●). The vertical bars denote the standard error of the mean. The experimental ED_50_ value (□) is significantly lower than the theoretical ED_50_ value (* *p* < 0.001, Student’s *t*-test), thus indicating a synergistic effect.

An isobologram was constructed for the combination treatment of paracetamol and β-caryophyllene oxide in a 1:1 fixed ratio (Figure 5). The isobolographic analysis found a theorical value of 64.16 ± 5.79 mg/kg for the ED_50_. The experimental ED_50_ of 45.3 ± 1.25 mg/kg is below the additive line, resulting in an interaction index of 0.70, which reflects a synergistic interaction.

### 3.3. Mechanism of Action of β-Caryophyllene Oxide 

Various pathways that mediate pain stemming from inflammation were analyzed as possible mechanisms of action of β-caryophyllene oxide.

#### 3.3.1. Participation of Opioid Receptors

**Figure 6:**
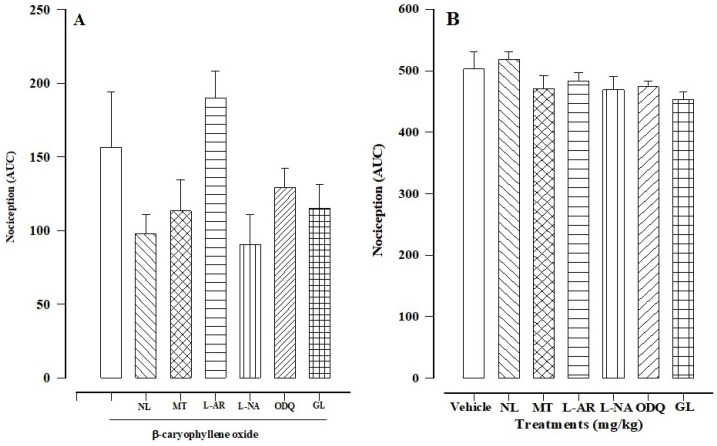
The mechanism of action of the anti-nociceptive effect of β-caryophyllene oxide (30 mg/kg) was explored by utilizing various pretreatments. The lower the AUC, the greater the percentage of anti-nociceptive activity. (**A**) The AUC of β-caryophyllene oxide when applied alone (at 30 mg/kg) or after any of the pretreatments: naloxone (NL, 5 mg/kg, i.p.), methiothepin (MT, 1 mg/kg, i.p.), l-arginine (l-AR, 100 mg/kg, i.p.), l-NAME (l-NA,10 mg/kg, i.p.), ODQ (0.1 mg/kg, i.p.), or glibenclamide (GL, 10 mg/kg, i.p.). Each bar portrays the average ± standard error of six mice per group. (**B**) The AUC of the vehicle control was not significantly different from that of the pretreatments administered alone (without β-caryophyllene oxide): naloxone (NL), methiothepin (MT), l-arginine (l-AR), l-NAME (l-NA), ODQ, or glibenclamide (GL). Each bar portrays the average ± standard error of the lesions of six animals.

Pretreatment with naloxone, a non-selective opioid receptor antagonist, slightly lowered the AUC value in phase II, reflecting a non-significant increase in the anti-nociceptive effect of β-caryophyllene oxide given at 30 mg/kg (80.54 ± 2.55% with pretreatment versus 68.90 ± 7.52% without pretreatment; Figure 6A). There was no significant difference between the response to the administration of naloxone alone and the response to the vehicle (control; Figure 6B).

#### 3.3.2. Contribution of Serotonin Receptors

Pretreatment with methiothepin, a non-selective serotonin receptor antagonist, slightly reduced the AUC value in phase II, reflecting a non-significant increase in the anti-nociceptive effect of β-caryophyllene oxide applied at 30 mg/kg (77.48 ± 4.22% with pretreatment versus 68.90 ± 7.52% without pretreatment; Figure 6A). There was no significant difference between the response to the administration of methiothepin alone and the response to the vehicle (control; Figure 6B).

#### 3.3.3. Participation of l-Arginine

Pretreatment with l-arginine, a NO precursor, slightly increased the AUC value in phase II, reflecting a non-significant decrease in the anti-nociceptive effect of β-caryophyllene oxide given at 30 mg/kg (62.25 ± 3.65% with pretreatment versus 68.90 ± 7.52% without pretreatment; Figure 6A). There was no significant difference between the response to the administration of l-arginine alone and the response to the vehicle (control; Figure 6B).

#### 3.3.4. Contribution of NO

Pretreatment with l-NAME, a NOS inhibitor, slightly decreased the AUC value in phase II, reflecting a non-significant increase in the anti-nociceptive effect of β-caryophyllene oxide applied at 30 mg/kg (82.03 ± 4.13% with pretreatment versus 68.90 ± 7.52% without pretreatment; Figure 6A). There was no significant difference between the response to the administration of l-NAME alone and the response to the vehicle (control; Figure 6B). 

#### 3.3.5. Participation of cGMP

The involvement of cGMP in the mechanism of action was examined by utilizing ODQ, a soluble guanylate cyclase inhibitor. Pretreatment with ODQ slightly decreased the AUC value in phase II, reflecting a non-significant increase in the anti-nociceptive effect of β-caryophyllene oxide given at 30 mg/kg (74.33 ± 2.58% with pretreatment versus 68.90 ± 7.52% without pretreatment; Figure 6A). There was no significant difference between the response to the administration of ODQ alone and the response to the vehicle (control; Figure 6B). 

#### 3.3.6. Contribution of the ATP-Sensitive K^+^ Channel 

The possible role of K^+^_ATP_ channels was assessed with glibenclamide, a blocker of K^+^_ATP_ sensitive channels. Pretreatment with glibenclamide slightly decreased the AUC value in phase II, reflecting a non-significant increase in the anti-nociceptive effect of β-caryophyllene oxide given at 30 mg/kg (77.15 ± 3.28% with pretreatment versus 68.90 ± 7.52% without pretreatment; Figure 6A). There was no significant difference between the response to the administration of glibenclamide alone and the response to the vehicle (control; Figure 6B). 

#### 3.3.7. The Effect of the Combination Treatment (Paracetamol + β-Caryophyllene Oxide) on the Gastric Mucosa

**Figure 7:**
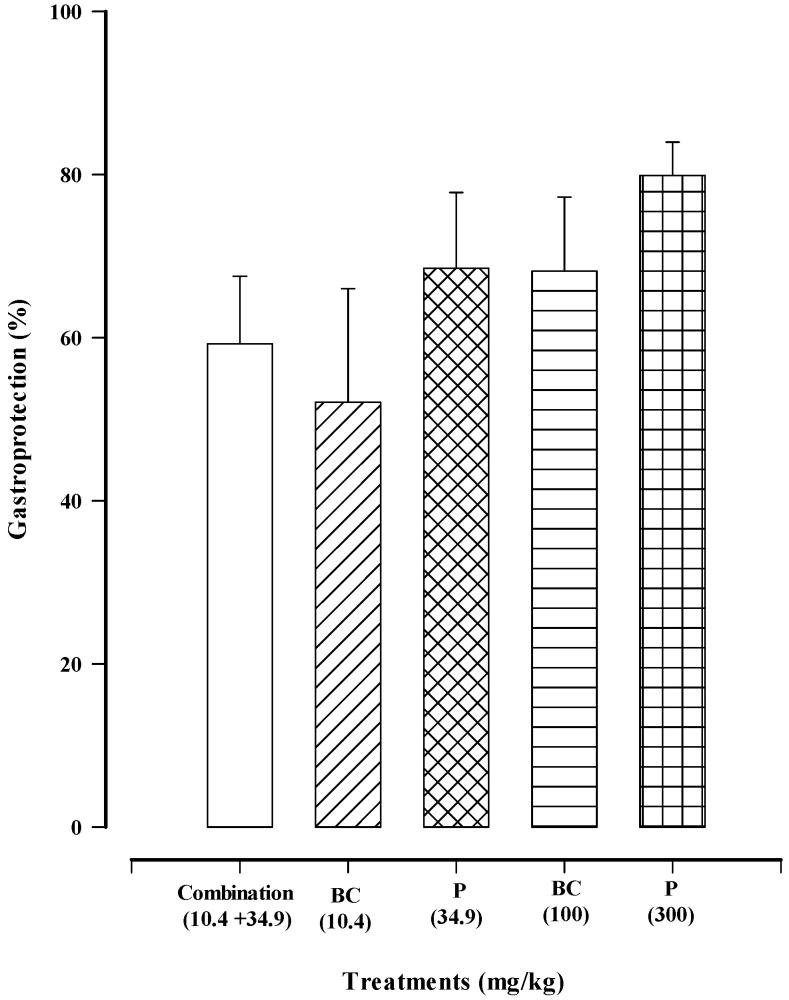
Gastroprotective effect (in the model of ethanol-induced gastric lesions) of the combination treatment of paracetamol (P) plus β-caryophyllene oxide (BC) and of the individual treatments with the same compounds. Each bar portrays the average ± standard error of the lesions of six animals.

The combination treatment (paracetamol at 34.9 mg/kg + β-caryophyllene oxide at 10.4 mg/kg) afforded 59.25 ± 8.3% gastroprotection against ethanol-induced gastric lesions. This result was not significantly different from the gastroprotection exhibited by the compounds when given individually (10.4 or 100 mg/kg of β-caryophyllene oxide; 34.9 or 300 mg/kg of paracetamol; Figure 7).

## 4. Discussion

Paracetamol is a popular analgesic and antipyretic agent with a weak anti-inflammatory effect [29]. The coadministration of paracetamol and β-caryophyllene oxide in a 1:1 ratio produced a dose-dependent anti-nociceptive effect in phase II of the formalin test, which is the procedure most commonly utilized for the assessment of pain and the preclinical evaluation of analgesic drugs [30]. In phase I of the formalin test, neurogenic pain is caused by the direct stimulation of nociceptors. Phase II is characterized by inflammation, involving both inflammatory mechanisms and central sensitization within the dorsal horn [30,31,32]. 

Both individual treatments (paracetamol and β-caryophyllene oxide) gave rise to a nociceptive effect during the development of the inflammatory processes of phase II, suggesting that they act by peripheral mechanisms. No significant activity was found in phase I, thus ruling out central mechanisms in the current study model. However, paracetamol has been reported to promote analgesia by acting at the central level when using other models [10]. For instance, experimental evidence from the hot plate test and the acetic acid-induced writhing test substantiates the role of β-caryophyllene oxide in central and peripheral pathways [16]. These discrepancies can be attributed to the use of distinct biological models [16,29,33,34].

The efficacy was similar when comparing β-caryophyllene oxide and paracetamol, as indicated by the similar maximum percentage of anti-nociception. However, the potency of β-caryophyllene oxide was 3.3-fold greater than that of paracetamol, as shown by the ED_50_ values. 

According to the isobolographic analysis, the combination of paracetamol and β-caryophyllene oxide generated a synergistic effect. Regarding drug combinations that provide a synergistic effect, the components usually act on different targets, thus impeding the progression of disease in distinct ways [35]. The synergism presently identified suggests that the mechanisms of action of paracetamol and β-caryophyllene oxide are at least partially different. 

The precise mechanism of action of paracetamol is still unknown [8]. It is reported to involve the inhibition of cyclooxygenases (COX-1, COX-2, and COX-3) and an interaction with the endocannabinoid system and serotonergic pathways. Moreover, it acts on transient receptor potential (TRP) channels and voltage-gated potassium (Kv7) channels, inhibits T-type calcium (Cav3.2) channels, and affects l-arginine in the NO synthesis pathway [8]. Paracetamol is metabolized to p-aminophenol and then converted to N-acylphenolamine (AM404), the most important mediator of analgesia. It is metabolized to other compounds as well, such as N-acetyl-p-benzoquinone imine (NAPQI), which also appears to produce analgesia [10]. 

Little is known about the anti-nociceptive mechanisms of action of β-caryophyllene oxide. The participation of cannabinoid receptors (CB1 or CB2) has been ruled out [12]. It has been proposed that β-caryophyllene oxide inhibits the release of inflammatory mediators of pain (e.g., TNF-α, PGE-2, COX-2, and IL-1β), but the mechanisms have not been defined [12].

Various pain-related pathways were presently evaluated to explore their relation to the nociceptive effect of β-caryophyllene oxide. One such pathway is related to opioid receptors, located in the central and peripheral nervous system. Their activation inhibits adenylyl cyclase and the production of cAMP. The direct interaction of cAMP with different membrane ion channels can modulate pre- and postsynaptic Ca^++^ currents and thereby attenuate the excitability of neurons and/or reduce the release of pronociceptive/proinflammatory neuropeptides. In addition, opioid receptor activation leads to the opening of G protein-coupled inwardly rectifying K^+^ (GIRK) channels, thus preventing neuronal excitation and/or propagation of action potentials [36]. Since the anti-nociceptive effect of β-caryophyllene oxide was not modified by pretreatment with naloxone (a non-selective opioid receptor antagonist), the corresponding mechanism of action in phase II of the formalin test did not involve opioid receptors.

The possible participation of serotonin (5-HT) receptors was also evaluated. Several studies have described the role of serotonin in regulating or inhibiting nociception pathways [37]. For example, a significant contribution has been reported of peripheral 5-HT2A, 5-HT3, and 5-HT7 receptors to peripheral nociceptive transmission during inflammation [38]. Hence, methiothepin (a non-selective antagonist of serotonin receptors) was used as a pretreatment before applying β-caryophyllene oxide. Since the effect of the latter compound was not significantly altered, serotonin receptors were not involved in its mechanism of action.

The peripheral anti-nociceptive effect of some drugs and natural products stems from the stimulation of the l-arginine-NO-sGC/cGMP/PKG/K^+^_ATP_ channel signaling pathway [39]. NOS enzymes oxidize l-arginine to L-citrulline, resulting in the release of NO. This in turn activates soluble guanylate cyclase (sGC), which leads to the production of cGMP, a second messenger that activates cGMP-dependent protein kinase (PKG). The latter activates multiple targets, such as the opening of K^+^_ATP_ channels, causing greater K^+^ current and thus a change in the intra- and extracellular K^+^ concentration, with the consequent depolarization or hyperpolarization inside and outside the membrane [40]. K^+^_ATP_ channels are known to be related in some cases to inflammatory processes, the regulation of nociception, and neuropathic pain [41]. 

To explore the possible contribution of the aforementioned signaling pathway, β-caryophyllene oxide was administered after various pretreatments: l-arginine (a NO precursor), l-NAME (a NOS inhibitor), ODQ (a soluble guanylate cyclase inhibitor), and glibenclamide (a blocker of K^+^_ATP_ sensitive channels). Since the anti-nociceptive effect of β-caryophyllene oxide was not modified by any of these pretreatments, l-arginine, NO, sGC, and K^+^_ATP_ channels were not involved in the mechanism of action of β-caryophyllene oxide in phase II of the formalin test. Although NO clearly had no role in the anti-nociceptive effect of β-caryophyllene oxide in the formalin model, it is known to take part in the gastroprotective mechanism of action of caryophyllene oxide, according to a report by our group [42].

In addition, β-caryophyllene oxide is known to reduce the release of inflammatory mediators of pain [12,31]. A previous study described the plausible contribution of prostaglandins in gastroprotection [42]. Therefore, future studies should examine the role of some inflammatory mediators, as they could be responsible, at least in part, for the synergistic effect of the combination treatment of paracetamol plus caryophyllene oxide.

There is a low incidence of adverse effects caused by paracetamol when compared to aspirin and other non-steroidal anti-inflammatory drugs [29]. Nevertheless, regular consumption can produce hypersensitivity reactions, kidney damage, anemia, thrombocytopenia, and gastrointestinal bleeding (at doses > 2 g taken chronically) [9]. 

Paracetamol in a single administration (at 80 or 500 mg/kg) is reported to diminish gastric damage induced in rats by some aggressive substances, such as ethanol, sodium hydroxide, and acidic aspirin [43,44]. Among the theories proposed to explain the gastroprotective effects of paracetamol are its possible activation of prostaglandin synthesis and the scavenging of free radicals, although the precise mode of action is not yet completely clear [45]. The dose of paracetamol in the combination treatment was much lower than the doses previously reported to provide a gastroprotective effect [43,44], perhaps because β-caryophyllene oxide also exerts a gastroprotective effect, as previously reported by our group in relation to rats [42]. Interestingly, β-caryophyllene oxide herein afforded a higher percentage of gastroprotection in mice than that previously identified in rats. According to the current results, the combination treatment of paracetamol plus β-caryophyllene oxide produced a strong anti-nociceptive effect in the formalin test and good gastroprotection against ethanol-induced gastric lesions. 

## 5. Future Prospects

It was found in the present study that the combination of β-caryophyllene oxide plus paracetamol generates a synergistic effect in the formalin model. To better understand this synergism, in the future, it will be necessary to explore the possible participation of other receptors in pain-related pathways. It would also be interesting to evaluate this same combination in other models of pain, such as neuropathic pain and the writhing test, to provide more in-depth knowledge of its potential. Moreover, the analgesic effect of the combination of paracetamol plus β-caryophyllene could be evaluated, given that the latter compound is a sesquiterpene structurally related to β-caryophyllene oxide and is known to have antinociceptive activity. Indeed, it is common to find β-caryophyllene and β-caryophyllene oxide together in diverse plants. On the other hand, the decreased dose of paracetamol in the combination treatment should reduce the adverse effects that can accompany chronic administration, such as hepatotoxicity. This possibility needs to be explored in the future.

## 6. Study Limitations

One of the limitations of this study was the testing of the antinociceptive activity of the combination treatment (β-caryophyllene oxide plus paracetamol) only in the formalin model. This allowed the investigation to maintain its focus on the possible synergism of the two compounds in the combination. In the future, the use of other pain models to test the antinociceptive activity of the combination treatment could possibly provide a more complete perspective on the role of β-caryophyllene oxide plus paracetamol under different conditions of pain. On the other hand, the current contribution determined the gastroprotective activity of the combination treatment to reduce the gastric damage that could be associated with chronic treatment. Other possible adverse effects of the administration of the combination treatment in the short and long run should be examined to know more about the risks and benefits of acute and chronic use.

## 7. Conclusions

The administration of the combination of paracetamol and β-caryophyllene oxide to mice gave rise to a synergistic analgesic effect in the formalin test. This characteristic of the combination treatment may be advantageous for the treatment of inflammatory pain in patients. Regarding the mechanism of action of the anti-nociceptive effect of β-caryophyllene oxide, the participation of opioid receptors, serotonin receptors, NO, and K^+^_ATP_ channels was ruled out. The co-administration of the two drugs protected against ethanol-induced gastric damage, which should help to reduce the possible adverse effects of chronic use.

## Figures and Tables

**Table 1:** Doses of paracetamol and β-caryophyllene oxide in the combination treatment (at a 1:1 ratio).

Combination	Doses of Paracetamol (mg/kg)	Doses of β-Caryophyllene Oxide (mg/kg)	Total (mg/kg)
1	98.17	29.67	127.84
2	49.08	14.84	63.92
3	24.54	7.42	31.96
4	12.27	3.71	15.98

## Data Availability

The datasets analyzed during the current study are available from the corresponding author upon reasonable request.
